# Gender-specific outcomes of deep brain stimulation for Parkinson’s disease — results from a single movement disorder center

**DOI:** 10.1007/s10072-023-06598-y

**Published:** 2023-01-06

**Authors:** Dorothee Kübler, Melanie Astalosch, Verena Gaus, Patricia Krause, Ana Luísa de Almeida Marcelino, Gerd-Helge Schneider, Andrea Kühn

**Affiliations:** 1 Movement Disorder and Neuromodulation Unit, Department of Neurology and Experimental Neurology, Charité - Universitätsmedizin Berlin, corporate member of Freie Universität Berlin and Humboldt-Universität zu Berlin, Hindenburgdamm 30, 12203 Berlin, Germany; 2 Department of Neurology and Experimental Neurology, Charité - Universitätsmedizin Berlin, corporate member of Freie Universität Berlin and Humboldt-Universität zu Berlin, Berlin, Germany; 3 Department of Neurosurgery, Charité - Universitätsmedizin Berlin, corporate member of Freie Universität Berlin and Humboldt-Universität zu Berlin, Berlin, Germany; 4 Berlin Center for Advanced Neuroimaging, Bernstein Center for Computational Neuroscience, Berlin, Germany; 5 Exzellenzcluster NeuroCure, Charité - Universitätsmedizin Berlin, Berlin, Germany; 6 Berlin School of Mind and Brain, Humboldt - Universität Zu Berlin, Berlin, Germany; 7 Deutsches Zentrum Für Neurodegenerative Erkrankungen, Berlin, Germany

**Keywords:** Parkinson’s disease, Deep brain stimulation, Gender, Personalized therapy, Non-motor symptoms, Outcome

## Abstract

**Introduction and goal:**

The investigation of gender differences in treatment response is crucial for effective personalized therapies. With only 30%, women are underrepresented in trials for deep brain stimulation (DBS) in Parkinson’s disease (PD). It is therefore important to evaluate gender-specific outcomes of DBS in PD in order to improve therapeutic counseling.

**Methods:**

We analyzed clinical outcome parameters of 203 patients with PD that underwent DBS surgery targeting the subthalamic nucleus (STN) at our movement disorder center. A total of 27.6% of patients were female and 72.4% male. Motor and non-motor scores were compared before and 1 year after DBS surgery (1y FU) using Wilcoxon signed-rank tests and gender specific outcomes were analyzed with chi-square tests.

**Results:**

At 1y FU, we found significant improvement in UPDRS II, UPDRS III (35.78 ± 36.14% MedOFF vs. StimON-MedOFF), UPDRS IV, depression (BDI-II), and health-related disability as (ADL) that showed no gender-specific differences. No significant change was revealed for UPDRS I, QUIP, and DemTect for the entire cohort. However, when analyzing both groups separately, only women improved in general cognition (plus 1.26 ± 3.03 DemTect points, *p* = 0.014*), whereas only men ameliorated in depression (minus 1.97 ± 6.92 BDI-II points, *p* = 0.002**) and impulsivity (minus 2.80 ± 7.27 QUIP points, *p* = 0.004**). Chi-square tests, however, revealed no significant differences between genders.

**Conclusion and outlook:**

STN-DBS is a highly effective treatment for motor and non-motor symptoms of PD for both women and men but our study hints towards gender-specific outcomes in non-motor-domains like cognition, depressive symptoms, and impulsivity. To explore this in more detail, larger cohorts need to be investigated in multicenter trials.

## Introduction

The investigation of sex and gender differences in diseases and their treatments is a crucial prerequisite for personalized medicine and effective therapies. The biological sex is differentiated from the term “gender” to also take into account sociocultural aspects. As both dimensions are meant in this paper, we limit the nomenclature to gender for reasons of easier legibility. Gender can influence different aspects of disease such as its psychosocial impact, response and adherence to treatment, communication between patients and medical staff, and coping strategies or disease management [1].

Many features of Parkinson’s disease (PD) display gender differences as comprehensively reviewed by Georgiev et al. [2]: Incidence (male-to-female ratio 1.2–1.5) and prevalence are slightly higher, and PD starts earlier in men. Women with PD show more tremor and less rigidity than men. Men are reported to show a higher disease burden [3]. In terms of non-motor symptoms, gender differences that are not PD-specific become apparent: Female PD patients perform better in tests of general cognition and verbal cognitive tasks and report more pain and more symptoms of depression than men. These disparities can also be seen in other diseases and in healthy individuals.

When it comes to therapy, a male predominance of PD patients undergoing deep brain stimulation (DBS) surgery of around 70% men [4] has been repeatedly reported in different populations. Shpiner et al. showed that of 207 PD patients referred for DBS surgery at the Miami University database, 76% were male. Of 100 patients who were consequently implanted with a DBS system, 77% were male. In female patients, personal preference was the main reason not to undergo DBS surgery whereas male patients predominantly failed to follow-up [5]. Postsurgical outcomes did not differ between women and men in this study. Subsequently, the authors advocate for better education in order to provide this highly effective treatment for all suitable candidates. On the other hand, following PD patients with STN-DBS up to 10 years, Andreasi et al. described a sustained motor effect in both sexes with female patients showing less clear long-term effects on bradykinesia and dyskinesia than men [6]. As personality and mood changes following DBS are one of the main concerns of PD patients, Dietrich et al. investigated 12 female and 34 male PD patients before as well as 1 and 2 years after DBS in the subthalamic nucleus (STN) [7]. Personality traits did not change significantly after surgery. Even though women scored higher than men on depressive symptoms at all timepoints, improvement in quality-of-life (QoL) after STN-DBS was higher in women than men. In most studies, although having a longer disease duration, women with PD improved more in activities of daily living (ADL) and QoL than men with DBS [2, 8]. Kim et al. investigated gender-specific motor and non-motor outcomes of STN-DBS after 1 and 5 years in 52 female and 48 male patients with PD [9]. No significant differences between women and men at both timepoints were found, except for an improvement in the physical components of health-related QoL in men after 5 years that was not seen in women.

Although gender differences in clinical presentation and treatment of PD are apparent, there are few studies systematically examining them. We investigated gender-specific postsurgical outcomes of STN-DBS in order to provide detailed knowledge on gender specific treatment response. The overarching goal is to provide personalized counseling for PD patients to make this highly effective treatment available for all suitable candidates.

## Methods

Patients with PD were included in this retrospective study if they have received DBS electrodes in the subthalamic nucleus between 01/2013 and 05/2020, and a follow-up visit had been performed 1 year after surgery (1y FU, 12.5 ± 2.8 months). All patients had undergone a thorough examination of diagnosis, indication, response to levodopa, and exclusion of possible contraindications prior to DBS surgery.

The following scales and scores were used preoperatively and at 1y FU: The Unified Parkinson’s Disease Rating Scale (UPDRS) was applied to characterize non-motor and motor aspects (UPDRS I and II) and motor complications (UPDRS IV) of the disease. UPDRS part III consists of the standardized motor examination that was conducted with and without dopaminergic medication preoperatively (MedON and MedOFF), and with and without STN-DBS postoperatively (StimON-MedOFF, StimON-MedON, StimOFF-MedOFF, and StimOFF-MedON). Symptoms of depression were monitored by means of the Beck Depression Inventory (BDI-II). The questionnaires for impulsive-compulsive disorders (QUIP) and the QUIP-rating scale (QUIP-RS) were used in order to screen for impulsive behaviors. Health-related disability was assessed by the German version of the Bain and Findley Activities of Daily Living (ADL) scale. Patients additionally completed the cognitive screening test DemTect assessing memory, language, visual construction, concentration, and executive function.

IBM SPSS Statistics, version 25 was used for calculation of all test results. Clinical characteristics are reported as mean ± standard deviation. Two-sided *p*-values < 0.05 were considered significant. Wilcoxon signed-rank tests were used to compare pre- and postoperative scores in the entire cohort as well as in women and men separately. Chi-square tests were conducted to evaluate statistically significant associations between genders in the proportion of patients concerning pre- and postoperative motor and non-motor scores and their changes following DBS.

## Results

In total, 229 patients with PD underwent DBS surgery at our movement disorder center between 01/2013 and 05/2020. Among them, 15 patients who received electrodes in the Ncl. ventralis intermedius of the thalamus and 11 implanted in the Globus pallidus internus were excluded from this study. The remaining 203 patients received electrodes targeting the STN and were included in this study (7 patients were implanted unilaterally).

A total of 27.6% of the included 203 PD patients with STN-DBS were female (*n* = 56) and 72.4% male (*n* = 147). Mean age was 60 ± 10 years with no difference between women and men. Demographic and clinical characteristics assessed preoperatively and at 1y FU and their relative change are shown in Table 1.

**Table 1:** Demographic and clinical characteristics preoperative and at 1y FU shown as mean, standard deviation (SD), and relative change (diff) for the whole cohort (all) and for women and men separately. # indicates patient numbers available for pre/post comparisons. Uncorrected *p*-values indicate significance levels of the comparison between pre- and postoperative scores (< 0.05 marked with * and < 0.005 marked with **)

		UPDRS I				UPDRS II				UPDRS III						
		pre	post	diff	p	pre	post	diff	*p*	MedOFF	MedON	MedOFF vs. MedON %	*p*	StimON-MedOFF	MedOFF vs. StimON-MedOFF %	*p*
All		#152	#119	#109		#152	#115	#106		#189	#193	#189		#127	#125	
	Mean	9.89	9.52	− 0.53	0.338	14.37	12.74	− 2.60	0.001**	45.29	22.59	49.46	< 0.001**	28.28	35.78	< 0.001**
	SD	6.58	6.09	6.97		7.75	7.93	9.10		17.55	11.97	19.94		16.91	36.14	
Women		#38	#35	#31		#38	#33	#29		#52	#54	#52		#38	#37	
	Mean	11.58	9.83	− 1.84	0.182	14.24	11.52	− 3.93	0.025*	43.50	20.09	51.73	< 0.001**	27.37	32.01	< 0.001**
	SD	6.58	5.45	7.60		7.78	7.15	8.34		20.45	10.19	17.02		11.97	41.98	
Men		#114	#84	#78		#114	#82	#77		#137	#139	#137		#89	#88	
	Mean	9.32	9.39	− 0.01	0.958	14.41	13.23	− 2.10	0.013*	45.96	23.55	48.60	< 0.001**	28.67	37.36	< 0.001**
	SD	6.51	6.37	6.68		7.78	8.21	9.38		16.35	12.50	20.93		18.68	33.52	

		UPDRS III					UPDRS IV				BDI-II				QUIP	
		StimON-MedOFF	MedOFFvs.StimONMedOFF%	*p*	StimOFF-MedOFF	StimOFFMedOFFvs. StimONMedOFF%	pre	post	diff	*p*	pre	post	diff	*p*	pre	post
All		#123	#122		#132	#107	#135	#95	#82		#175	#126	#122		#146	#102
	Mean	49.00	42.18	< 0.001**	20.58	39.89	7.82	3.94	− 4.29	< 0.001**	12.43	10.83	− 1.76	0.004**	5.63	3.67
	SD	19.22	22.86		12.11	17.78	5.15	4.19	6.14		7.55	6.71	7.40		7.45	5.36
Women		#39	#38		#37	#33	#36	#30	#25		#48	#36	#35		#38	#32
	Mean	44.67	37.36	< 0.001**	20.08	37.03	7.72	4.13	− 3.40	0.023*	12.88	11.86	− 1.26	0.471	5.24	5.28
	SD	17.52	20.94		12.22	17.69	5.17	3.97	6.22		6.94	7.07	8.58		9.57	6.63
Men		#84	#84		#95	#74	#99	#65	#57		#127	#90	#87		#108	#70
	Mean	51.01	44.37	< 0.001**	20.77	41.16	7.86	3.85	− 4.68	< 0.001**	12.26	10.42	− 1.97	0.002**	5.77	2.93
	SD	19.74	23.47		12.12	17.80	5.17	4.31	6.12		7.78	6.55	6.92		6.60	4.52

		QUIP		QUIP-RS				ADL				DemTect				
		diff	*p*	pre	post	diff	*p*	pre	post	diff	*p*	pre	post	diff	*p*	
All		#86		#148	#111	#95		#161	#96	#86		#158	#94	#80		
	Mean	− 1.80	0.059	10.90	7.12	− 3.22	0.047*	17.96	14.05	− 5.70	< 0.001**	14.33	14.45	0.64	0.131	
	SD	9.11		13.27	9.09	15.33		13.14	11.50	12.52		3.08	3.80	3.70		
Women		#27		#38	#33	#28		#46	#28	#27		#46	#29	#27		
	Mean	0.37	0.542	9.89	9.91	0.75	0.626	19.74	14.04	− 6.15	0.036*	14.52	15.66	1.26	0.014*	
	SD	12.10		16.58	11.96	20.34		13.66	9.46	12.63		2.81	2.83	3.03		
Men		#59		#110	#78	#67		#115	#68	#59		#112	#65	#53		
	Mean	− 2.80	0.004**	11.25	5.94	− 4.88	0.004**	17.24	14.06	− 5.49	0.001**	14.25	13.91	0.32	0.598	
	SD	7.27		11.98	7.34	12.50		12.91	12.30	12.57		3.19	4.07	3.99		

### DBS outcomes of the entire cohort at 1y FU

**Fig. 1:**
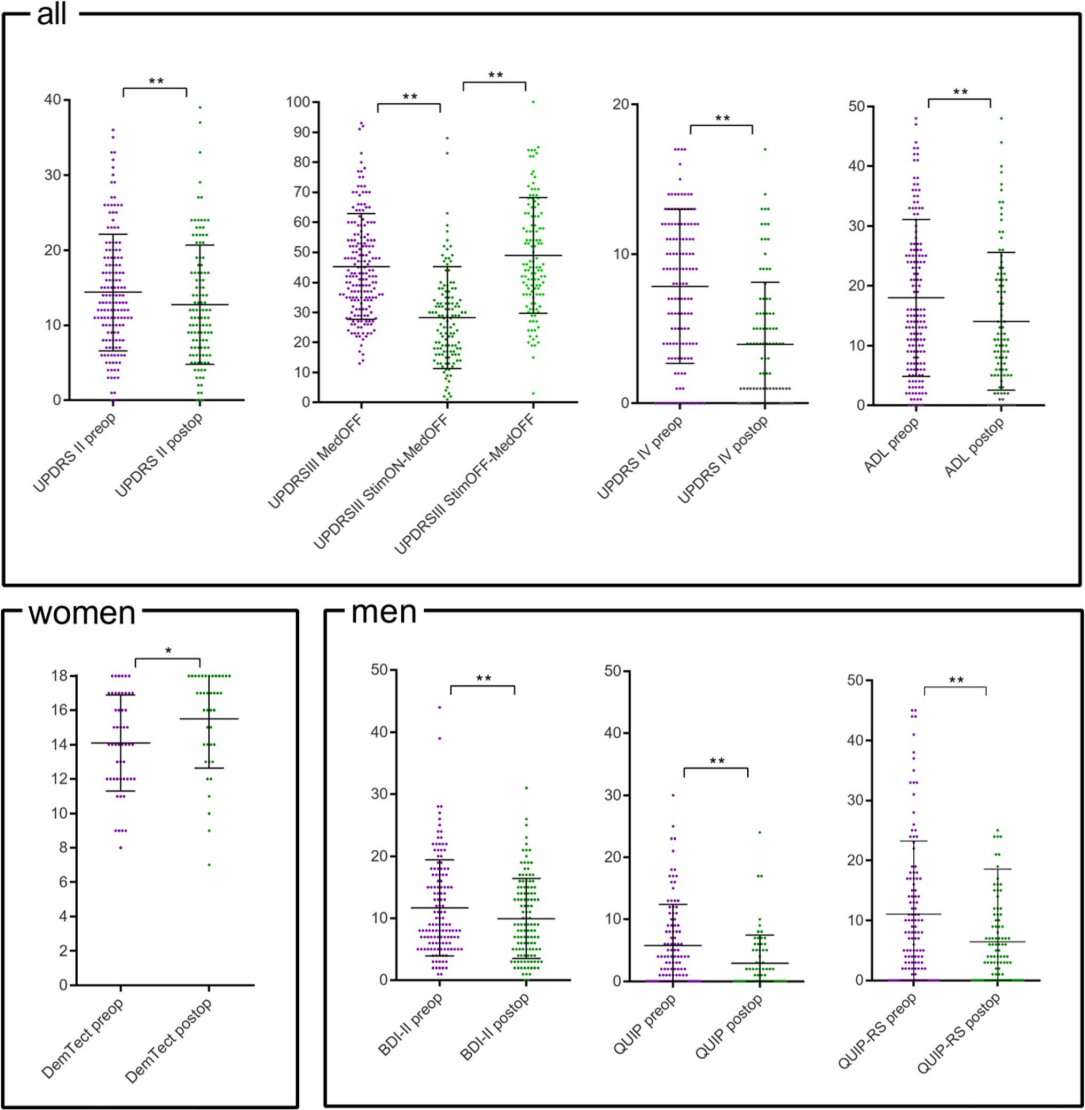
Pre- and postoperative scales and scores with significant differences pre- vs. postoperatively of the whole cohort (all) and analyzed for women and men separately. Significance levels are marked with * for *p*-values ≤ 0.05 and with ** for *p*-values ≤ 0.005

At 1y FU, motor impairment as measured by the UPDRS III score improved significantly with DBS (MedOFF vs. StimON-MedOFF: relative mean improvement 35.78 ± 36.14%, *p* < 0.001**; StimOFF-MedOFF vs. StimON-MedOFF: relative mean improvement 42.18 ± 22.86%, *p* < 0.001**). For visualization, please see Fig. 1. The UPDRS IV indicating severity of motor fluctuations improved significantly as well (absolute mean improvement − 4.29 ± 6.14 points, *p* < 0.001**). Health-related disability as measured by the ADL (absolute mean improvement − 5.70 ± 12.52 points, *p* < 0.001**) as well as non-motor symptoms as measured by the UPDRS II (absolute mean improvement − 2.60 ± 9.10 points, *p* = 0.001**) and depression as measured by the BDI-II (absolute mean improvement − 1.76 ± 7.40 points, *p* = 0.004*) were significantly ameliorated. No significant change was revealed for UPDRS I, QUIP, and DemTect.

### DBS outcomes at 1 yr FU analyzed separately for women and men

Only women (DemTect absolute mean improvement 1.26 ± 3.03 points, *p* = 0.014*) but not men (− 0.32 ± 3.99, *p* = 0.598) improved in the cognitive screening test after STN-DBS. Significance levels can also be seen in Table 1.

Self-ratings of depressive symptoms were significantly ameliorated at 1y FU in men (BDI-II absolute mean improvement − 1.97 ± 6.92 points, *p* = 0.002**) but not in women (− 1.26 ± 8.58, *p* = 0.471). Men also showed a significant reduction of impulsivity as measured by the QUIP (absolute mean improvement − 2.80 ± 7.27 points, *p* = 0.004**) and QUIP-RS (absolute mean improvement − 4.88 ± 12.50 points, *p* = 0.004**) but women did not (0.37 ± 12.10, *p* = 0.542 and 0.75 ± 20.34, *p* = 0.626 respectively).

These analyses were explorative and were not corrected for multiple comparisons. Chi-square tests taking into account the proportion of men and women within the cohort revealed no significant differences between genders in the pre- and postoperative scales or in the relative change of these scales at 1 y FU.

## Discussion

STN-DBS is a highly effective treatment of PD for both women and men. In this retrospective study, both genders responded well to STN-DBS in terms of motor symptoms and motor fluctuations. Concerning non-motor symptoms, overall symptom severity and health-related disability could significantly be reduced as well. Chi-square tests revealed no significant differences between men and women in the pre- and postoperative scales or in the relative improvement of these scales at 1y FU. These findings are similar to those of previous studies although our cohort was the largest sample of PD patients with STN-DBS analyzed regarding gender-specific outcomes to date. As our study cohort consists of a defined pre-selected sample of PD patients that were considered suitable for DBS surgery and therapy, we focused on therapy outcomes rather than preoperative differences of women and men. The lack of significant postoperative gender differences most likely constitutes an effect of under-representation of women as they constitute only one third of patients, so gender effects are difficult to verify.

When looking into DBS outcomes of non-motor symptoms separately in the two cohorts using an exploratory approach, gender-specific differences could be revealed: At 1y FU, only women showed an improvement in general cognitive abilities whereas in men, symptoms of depression and impulsivity could be reduced. Interestingly, Hariz et al. also described an improvement in cognition and additionally greater ADL amelioration following DBS specifically in women with PD [10]. Göttgens et al. summarized that although physical complaints are similar in female and male patients with PD, the psychological burden seems to show a gender-specific pattern: Women suffer more from changes in their intimate relationships whereas men have more difficulties with self-presentation, concepts that are not specific to PD. The authors stress that these aspects should be attributed to feminine or masculine gender roles and are not a consequence of gender per se.

The most obvious gender difference, however, is that women with PD are less likely to undergo DBS, which cannot solely be explained by the slightly higher number and younger onset of male PD patients. Interestingly, a similar male predominance is reported in DBS for Essential Tremor [11], a disease with a similar lifetime prevalence in women and men. Vlaanderen et al. found that women suffer from complications earlier during the course of PD and have contact to the healthcare system sooner and more often than men [12]. Possible reasons for the gender discrepancy in DBS surgery include factors both on the provider and on the patient side. Jost et al. report a disproportionate bias in referrals for DBS evaluation with only 30% female PD patients that are more likely to actually receive DBS surgery when referred than men [13]. Nevertheless, also in this study, women with PD profited as much from this treatment than men. Unfortunately, for the period of the current study, data regarding referrals for DBS evaluation and reasons not to undergo surgical treatment were not available. Therefore, with our data, we cannot infer on reasons why women with PD are underrepresented in DBS treatment. However, in patients evaluated for DBS surgery on our neurological ward between 06/2019 and 06/2021, women were no more likely to decline or to be dissuaded from surgery than men. In order to detect a possible unconscious bias in counseling in favor or against DBS with respect to a patient’s gender, the reasons for referrals and the gender of the referring physicians are interesting questions for further studies. Hamberg et al. investigated gender specific decision-making patterns concerning DBS surgery from the patient perspective [14]: Male PD patients were more likely to proactively take their own initiative or agreed when DBS was offered whereas female PD patients were more fearful of complications and consulted their family and friends more often. In addition, more men were professionally active when DBS surgery was performed and undertaking active steps towards DBS surgery was associated with leadership experience. Interestingly, Vinke et al. report a significant increase of female PD patients undergoing STN-DBS (from 17 to 42%) after changing their operative technique from intraoperative wakefulness during surgery to general anesthesia [15]. In sum, there seems to be both a referral and a request bias leading to the gender discrepancy in DBS surgery. In their recent paper, Subramanian et al. provide suitable strategies to encounter gender bias in PD management [16], both on the patient and the caregiver side.

It is important to acknowledge the limitations of this study. In this regard, we primarily have to mention the study’s single center retrospective design, the relatively short time of follow-up and the fact that not all scores and scales were available for all patients. Another important constraint to be discussed is the definition of gender derived from patient records describing patients as either female or male. As different concepts of self-identification were not systematically asked for, no inferences can be made on sexual and gender minorities [17].

In our opinion, personalized therapeutical counseling has to take gender aspects into account in order to provide effective treatment strategies for all suitable candidates. Therefore, we aim to further investigate gender differences of DBS outcomes in an international multicenter study.
