# Correction to: Gender‑specific outcomes of deep brain stimulation for Parkinson’s disease — results from a single movement disorder center

**DOI:** 10.1007/s10072-023-06661-8

**Published:** 2023-02-07

**Authors:** Dorothee Kübler, Melanie Astalosch, Verena Gaus, Patricia Krause, Ana Luísa de Almeida Marcelino, Gerd-Helge Schneider, Andrea Kühn

**Affiliations:** 1 Movement Disorder and Neuromodulation Unit, Department of Neurology and Experimental Neurology, Charité - Universitätsmedizin Berlin, corporate member of Freie Universität Berlin and Humboldt-Universität zu Berlin, Hindenburgdamm 30, 12203 Berlin, Germany; 2 Department of Neurology and Experimental Neurology, Charité - Universitätsmedizin Berlin, corporate member of Freie Universität Berlin and Humboldt-Universität zu Berlin, Berlin, Germany; 3 Department of Neurosurgery, Charité – Universitätsmedizin Berlin, corporate member of Freie Universität Berlin and Humboldt-Universität zu Berlin, Berlin, Germany; 4 Berlin Center for Advanced Neuroimaging, Bernstein Center for Computational Neuroscience, Berlin, Germany; 5 Exzellenzcluster NeuroCure, Charité – Universitätsmedizin Berlin, Berlin, Germany; 6 Berlin School of Mind and Brain, Humboldt - Universität Zu Berlin, Berlin, Germany; 7 Deutsches Zentrum Für Neurodegenerative Erkrankungen, Berlin, Germany

**Correction to: Neurological Sciences (2023)**

10.1007/s10072-023-06598-y

The above article was published with error in author names. The correct capturing of the author name “Ana Luísa de Almeida Marcelino” has been updated.

The Original article has been corrected.

